# Prevalence of stillbirth and its associated factors in East Africa: generalized linear mixed modeling

**DOI:** 10.1186/s12884-021-03883-6

**Published:** 2021-06-02

**Authors:** Getayeneh Antehunegn Tesema, Zemenu Tadesse Tessema, Koku Sisay Tamirat, Achamyeleh Birhanu Teshale

**Affiliations:** Department of Epidemiology and Biostatistics, Institute of Public Health, College of Medicine and Health Sciences, University of Gondar, Gondar, Ethiopia

**Keywords:** Stillbirth, East Africa, Mixed-effect analysis, DHS

## Abstract

**Background:**

Stillbirth is the most frequently reported adverse pregnancy outcome worldwide, which imposes significant psychological and economic consequences to mothers and affected families. East African countries account for one-third of the 2.6 million stillbirths globally. Though stillbirth is a common public health problem in East African countries, there is limited evidence on the pooled prevalence and associated factors of stillbirth in East Africa. Therefore, this study aimed to investigate the prevalence of stillbirth and its associated factors in East Africa.

**Methods:**

This study was based on the most recent Demographic and Health Surveys (DHSs) of 12 East African countries. A total weighted sample of 138,800 reproductive-age women who gave birth during the study period were included in this study. The prevalence of stillbirth with the 95% Confidence Interval (CI) was reported using a forest plot. A mixed-effect binary logistic regression analysis was done to identify significantly associated factors of stillbirth. Since the DHS data has hierarchical nature, the presence of clustering effect was assessed using the Likelihood Ratio (LR) test, and Intra-cluster Correlation Coefficient (ICC), and deviance were used for model comparison. Variables with a p-value of less than 0.2 in the bi-variable analysis were considered for the multivariable analysis. In the multivariable mixed-effect binary logistic regression analysis, the Adjusted Odds Ratio (AOR) with 95% CI were reported to declare the strength and significance of the association.

**Results:**

The prevalence of stillbirth in East Africa was 0.86% (95% CI: 0.82, 0.91) ranged from 0.39% in Kenya to 2.28% in Burundi. In the mixed-effect analysis; country, women aged 25–34 years (AOR = 1.27, 95% CI: 1.11, 1.45), women aged ≥ 35 years (AOR = 1.19, 95% CI: 1.01, 1.44), poor household wealth (AOR = 1.07, 95% CI: 1.02, 1.23), women who didn’t have media exposure (AOR = 1.11, 95% CI: 1.01, 1.25), divorced/widowed/separated marital status (AOR = 2.99, 95% CI: 2.04, 4.39), caesarean delivery (AOR = 1.81, 95% CI: 1.52, 2.15), preceding birth interval < 24 months (AOR = 1.15, 95% CI: 1.06, 1.24), women attained secondary education or above (AOR = 0.68, 95% CI: 0.56, 0.81) and preceding birth interval ≥ 49 months (AOR = 1.45, 95% CI: 1.28, 1.65) were significantly associated with stillbirth.

**Conclusions:**

Stillbirth remains a major public health problem in East Africa, which varied significantly across countries. These findings highlight the weak health care system of East African countries. Preceding birth interval, county, maternal education media exposure, household wealth status, marital status, and mode of delivery were significantly associated with stillbirth. Therefore, public health programs enhancing maternal education, media access, and optimizing birth spacing should be designed to reduce the incidence of stillbirth.

## Background

Stillbirth remains a huge challenge in low-and middle-income countries particularly in East African countries [1]. For international comparison, the World Health Organization (WHO) defines stillbirth as a baby born with no signs of life at or after 28 weeks gestation or birth weight of ≥ 1000 g [2]. An estimated 2.6 million stillbirths occurred globally per year [3], of these, the vast majority (98%) occurred in low and middle-income countries [3, 4] with three-fourths occurred in south Asia and sub-Saharan Africa (SSA) [5]. Nearly 60% of stillbirths occurred in rural families since they are often the poorest with limited access to midwifery care, family planning services, and emergency obstetric care such as caesarean section [6, 7].

More than half of all stillbirths occurred during labor and the majority of stillbirths can be preventable by accessing maternal healthcare [1, 8, 9]. Despite the remarkable reduction in global SBR from 24.9 per 1000 births in 2000 to 18.9 per 1000 live births in 2015 with an Annual Rate of Reduction (ARR) of 2% [4, 10], it has been slower than maternal, neonatal and child mortality particularly in SSA [11]. However, developed countries have shown substantial progress in reducing stillbirths, East African countries are far below to achieve the Every Newborn Action Plan (ENAP) target of reducing Stillbirth Rates (SBR) of 12 per 1000 births or less by 2030 [4, 12].

In developing countries; maternal infection, fetal asphyxia, trauma, congenital abnormalities, fetal-maternal hemorrhage, prolonged labor, fetal distress, and congenital infections and underlined maternal medical conditions were significantly contributed to stillbirths [13–15]. Most countries including East African countries didn’t t include stillbirth in their vital statistics reporting system, and it remains invisible and underreported [2, 16]. The available literature identified pregnancy and health service-related factors, socio-demographic and economic factors as significant predictors of stillbirth in developing countries. Potential maternal obstetric related factors include parity [17], preceding birth interval [18], multiple gestation [19], mode of delivery [20], place of delivery [21], wanted pregnancy [22], and Antenatal Care (ANC) visit [23]. Socio-demographic and economic factors that have a significant correlation with stillbirth are maternal age [24], maternal education [25], household wealth status [26], residence [27], marital status [28], and husband education [29].

Pregnancies from mothers with poverty and poor education are more likely to be stillborn since poverty is closely linked with poor maternal health care services utilization and food insecurity [30]. Stillbirth has significant health and economic consequences [31]. Mothers face psychological effects after stillbirth, including anxiety and depression, post-traumatic stress disorder, and stigmatization [32, 33].

The incidence significantly varied across regions with the highest SBR documented in East African countries [34, 35]. Despite the vast majority of stillbirths occurring in Sub-Saharan Africa (SSA), as to our search of the literature, there is no study done on the pooled prevalence and associated factors of stillbirth in East Africa. To remarkably reduce stillbirth rates in East African countries, factors that contribute to the increased risk of experiencing stillbirth among pregnant women are crucial for achieving the ambitious targets of ENAP by 2030. Therefore, this study aimed to investigate the pooled prevalence and associated factors of stillbirth in East Africa. Investigating the factors influencing stillbirth will facilitate the development of better public health interventions to reduce these preventable deaths and to improve maternal health.

## Methods

### Data sources

The data source for this study was the Demographic and Health Survey (DHS) data of 12 East countries (Burundi, Ethiopia, Comoros, Uganda, Rwanda, Tanzania, Mozambique, Madagascar, Zimbabwe, Kenya, Zambia, and Malawi). The DHS is a nationally representative survey that collects data on basic health indicators like mortality, morbidity, family planning service utilization, fertility, maternal and child health services (vaccination). The data of each country was derived from the measure DHS program. Each country's DHS survey consists of different datasets including men, women, children, birth, and household datasets; for this study, we used the Birth Record dataset (BR file). In the BR file, all births after 7 months of gestation in the last five years preceding the survey were interviewed. The datasets of 12 East African countries were appended together to determine the pooled prevalence of stillbirth and associated factors in East Africa. The DHS employed a two-stage stratified sampling technique to select the study participants. In the first stage, Enumeration Areas (EAs) were randomly selected while in the second stage households were selected. We pooled 12 DHS surveys done in the 12 East African countries, and a total weighted sample of 138,800 births after 7 months of gestation were included in the study (Table 1).

**Table 1:** Countries year of survey and sample size

Country	Year of survey	Unweighted sample	Weighted sample
Burundi	2016/17	13,192	13,611
Ethiopia	2016	10,641	11,023
Kenya	2014	20,964	19,563
Comoros	2012	3149	3235
Madagascar	2008	12,448	12,686
Malawi	2011	17,286	17,395
Mozambique	2011	11,102	11,704
Rwanda	2014/15	7856	8003
Tanzania	2015/16	10,233	10,052
Uganda	2016	15,522	15,270
Zambia	2018/19	9959	9841
Zimbabwe	2015	6132	6418

### Study variables

#### Outcome variable

The 2016 EDHS asked women to report any pregnancy loss that occurred in the last five years preceding the survey. The duration of pregnancy was reported for every pregnancy separately which did not result in a live birth. Pregnancy losses occurring after seven completed months of gestation were considered as stillbirth (28). The response variable for this study was the occurrence of stillbirth among mothers of childbearing age (15–49 years). The response variable for the ith mother was represented by a random variable Yi with two possible values coded as 1 and 0. So, the response variable of the ith mother Yi was measured as a dichotomous variable with possible values Yi = 1, if ith mother had experienced stillbirth and Yi = 0 if the mother had a live birth.

### Independent variables

Socio-demographic and economic variables, maternal obstetric, and health service-related variables were included as independent variables. Socio-demographic and economic variables considered were residence (recoded as rural and urban), country, maternal education status (recoded as no education, primary education, and secondary education and above), husband education status (recoded as no education, primary education, secondary education and above), maternal age (recoded as 15–24 years, 25–34 years and 35–49 years), maternal occupation (recoded as no and yes), household wealth status (recoded as poor, middle and rich), marital status (recoded as single, married, and divorced/widowed/separated), and media exposure (recoded as no and yes). The maternal obstetric and health service-related variables included were parity (recoded as one, two to four, and five and above), place of delivery (home and health facility), mode of delivery (recorded as vaginal, and caesarean delivery), covered by health insurance (recoded as no and yes), number of ANC visit (recoded as no ANC visit, 1–3 ANC visit and ≥ 4 ANC visit) and preceding birth interval (recoded as less than 24 months, 25–48 months and ≥ 49 months).

### Data management and analysis

We pooled the DHS data of 12 East African countries together after extracting the variables based on literature. Before any statistical analysis was conducted, the data were weighted using sampling weight, primary sampling unit, and strata to restore the representativeness of the survey and take sampling design when calculating standard errors and reliable estimates. "Svy set" STATA command was used for the descriptive analysis to take into account the complex survey design. Cross tabulations and summary statistics were done using STATA version 14 software. The pooled prevalence of stillbirth with the 95% Confidence Interval (CI) was reported using a forest plot.

The DHS data had a hierarchical nature, this could violate the independence of observations and equal variance assumption of the traditional logistic regression model. Hence, women are nested within a cluster, we expect that women within the same cluster are more likely to be related to each other than women in another cluster. This implies that there is a need to take into account the between cluster variability by using advanced models. Therefore, for the associated factors, we used the mixed-effect logistic regression model. The presence of clustering effect was assessed using Intra-class Correlation Coefficient (ICC), and Likelihood Ratio (LR) test. Deviance (-2LLR) was used for model comparison since the models were nested. Accordingly, a mixed effect logistic regression model (both fixed and random effect) was the best-fitted model since it had the lowest deviance value. Variables with a *p*-value < 0.2 in the bi-variable analysis were considered in the multivariable mixed-effect logistic regression model. Adjusted Odds Ratios (AOR) with a 95% Confidence Interval (CI) and p-value ≤ 0.05 in the multivariable model were used to declare significant factors associated with stillbirth.

### Ethical consideration

Ethical approval and participant consent were not necessary for this particular study since the study was a secondary data analysis based on the publicly available DHS data from the MEASURE DHS program. We requested the data from the MEASURE DHS Program and permission was granted to download and use the data for this study from http://www.dhsprogram.com. There are no names of individuals or household addresses in the data files.

## Results

Socio-demographic and economic characteristics of the study participants.

A total of 138,800 reproductive-age women who gave birth during the study period were included. The median age of respondents was 28 years (IQR ± 5). Of the total, 108,692 (78.3%) of the women were residing in rural areas and 19,563 (14.1%) were from Kenya. The majority (53.2%) of the women were attained primary education and 42.4% of their husbands were attained secondary education and above. About 63,093 (45.5%) of the women were from poor households (Table 2).

**Table 2:** The socio-demographic and economic characteristics of the women who gave birth during the study period in the 12 East African countries

Variable	Frequency	Percentage (%)
**Residence**		
Urban	30,108	21.7
Rural	108,692	78.3
**Country**		
Burundi	13,611	9.8
Ethiopia	11,023	7.9
Kenya	19,563	14.1
Comoros	3233	2.3
Madagascar	12,685	9.1
Malawi	17,395	12.5
Mozambique	11,704	8.4
Rwanda	8003	5.8
Tanzania	10,052	7.2
Uganda	15,270	11.0
Zambia	9841	7.1
Zimbabwe	6418	4.6
**Maternal education status**		
No	33,448	24.1
Primary education	73,808	53.2
Secondary and above	31,544	22.7
**Husband education status**		
No	22,652	16.3
Primary	57,352	41.3
Secondary and above	58,796	42.4
**Maternal age (in years)**		
15–24	41,683	30.0
25–34	66,067	47.6
≥ 35	31,050	22.4
**Maternal occupation**		
No	44,615	32.1
Yes	94,185	67.9
**Wealth status**		
Poor	63,093	45.5
Middle	26,820	19.3
Rich	48,887	35.2
**Marital status**		
Single	97,513	70.3
Married	20,977	15.1
Divorced/widowed/separated	20,190	14.6
**Media exposure**		
No	48,700	35.1
Yes	90,100	64.9

### Maternal obstetric and health service-related characteristics of the study population

About 106,632 (76.8%) of the women were giving birth at home and 130,515 (94.0%) of the birth were vaginal delivery. Three-fourth (66.9%) of the women had 4 and above ANC visits during pregnancy. More than half (51.7%) of the women had 5 and above births (Table 3).

**Table 3:** The maternal obstetric and health service related characteristics of the women who gave birth in the 12 East African countries

Variable	Frequency	Percentage (%)
**Parity**		
1	22,172	16.0
2–4	71,730	51.7
≥ 5	44,898	32.3
**Place of delivery**		
Home	38,002	27.4
Health facility	100,798	72.6
**Mode of delivery of the last child**		
Vaginal	130,515	94.0
Caesarean delivery	8285	6.0
**Covered by health insurance**		
No	106,632	76.8
Yes	32,168	23.2
**ANC visit**		
No visit	6125	4.4
1–3	39,791	28.7
≥ 4	92,884	66.9
**Preceding birth interval**		
< 24	18,962	13.7
25–48	58,589	42.2
> 48	61,249	44.1

### Prevalence of stil birth in East Africa

**Fig. 1:**
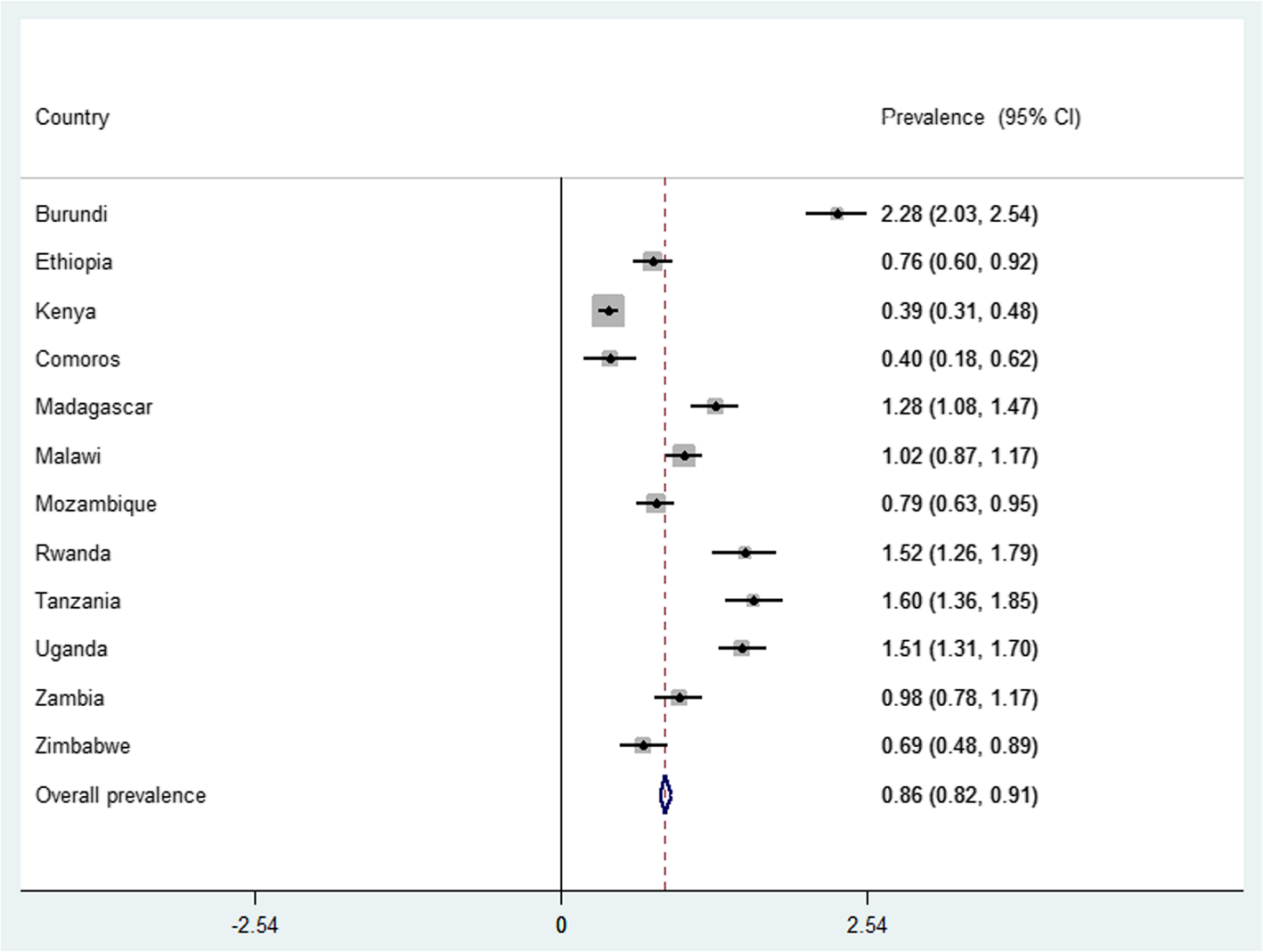
The prevalence of stillbirth in East Africa

The pooled stillbirth rates in East Africa was 0.86% (95% CI: 0.82%, 0.91%) with significant variation across countries ranged from 0.39% in Kenya to 2.28% in Burundi (Fig. 1).

### Factors associated with stillbirth

#### Model comparison

AIC, BIC, and deviance were checked and reported as a model comparison parameter. Since the models were nested models we preferred deviance value for model comparison and the mixed effect logistic regression model was the best-fitted model because of the smallest value of deviance (Table 4). Furthermore, the ICC value which was 0.63 (0.46, 0.86), indicates that about 63% of the overall variability in stillbirth was explained by the difference across clusters while the remaining 37% was attributable to the individual difference. The Log-likelihood ratio test which was (X ^2^ = 66.36, *p*-value < 0.001) informed us to choose a mixed-effect logistic regression model (GLMM) over the basic model.

**Table 4:** model comparison between standard logistic regression and mixed-effect logistic regression analysis

Parameter	Deviance	AIC	BIC
Standard logistic regression analysis	53,812.96	53,878.96	54,203.63
Mixed-effect logistic regression analysis	53,665.16	53,733.15	54,067.66

In the multivariable mixed-effect logistic regression analysis; country, maternal age, media exposure, wealth status, marital status, mode of delivery, maternal education status, and preceding birth interval were significantly associated with stillbirth. The odds of experiencing stillbirth among women in Burundi, Ethiopia, Madagascar, Malawi, Mozambique, Rwanda, Tanzania, Uganda, Zambia and Zimbabwe were 5.41 (AOR = 5.41, 95% CI: 4.17, 7.02), 2.35 (AOR = 2.35, 95% CI: 1.71, 3.22), 3.21 (AOR = 3.21, 95% CI: 2.44, 4.22), 2.13 (AOR = 2.13, 95% CI: 1.61, 2.80), 2.25 (AOR = 2.25, 95% CI: 1.65, 3.07), 3.22 (AOR = 3.22, 95% CI: 2.41, 3.41), 4.89 (AOR = 4.89, 95% CI: 3.76, 6.36), 4.10 (AOR = 4.10, 95% CI: 3.17, 5.30), 3.20 (AOR = 3.20, 95% CI: 2.38, 4.31), and 2.13 (AOR = 2.13, 95% CI: 1.47, 3.08) times higher than women living in Kenya, respectively. Women aged 25–34 years and ≥ 35 years were 1.27 (AOR = 1.27, 95% CI: 1.11, 1.45) and 1.19 (AOR = 1.19, 95% CI: 1.01, 1.44) times higher odds of experiencing stillbirth compared to women aged 15–24 years, respectively. The odds of experiencing stillbirth among women in the poor household were 1.07 (AOR = 1.07, 95% CI: 1.02, 1.23) higher than women in the rich household. Mothers who didn’t have media exposure had 1.11 times (AOR = 1.11, 95% CI: 1.01, 1.25) higher odds of stillbirth compared to mothers who had media exposure. The odds of experiencing stillbirth among divorced/widowed/separated women and single were 2.99 (AOR = 2.99, 95% CI: 2.04, 4.39) and 3.24 (AOR = 3.24, 95% CI: 2.27, 4.63) times higher than single women. The odds of stillbirth among birth who gave birth through caesarean section were 1.81 times (AOR = 1.81, 95% CI: 1.52, 2.15) than vaginal delivery. Mothers who had a birth interval of fewer than 24 months and ≥ 49 months were 1.15 (AOR = 1.15, 95% CI: 1.06, 1.24) and 1.45 (AOR = 1.45, 95% CI: 1.28, 1.65) times higher odds of experiencing stillbirth than mothers who had birth interval 24 – 48 months respectively. The odds of experiencing stillbirth among mothers who attained secondary education or above were decreased by 32% (AOR = 0.68, 95% CI: 0.56, 0.82) (Table 5).

**Table 5:** The bi-variable and multivariable mixed effect logistic regression analysis of stillbirth among reproductive age women in the 12 East African countries

Variable	Outcome of birth		Crude Odds Ratio (COR) with 95% CI	Adjusted Odds Ratio (AOR) with 95% CI
	**live birth**	**Stillbirth**		
**Residence**				
Urban	29,872	236	1	1
Rural	107,359	1335	1.53 (1.35, 1.75)	1.29 (0.98, 1.54)
**Country**				
Kenya	19,487	77	1	1
Burundi	13,300	311	5.66 (4.42, 7.26)	5.41 (4.17, 7.02)^*^
Ethiopia	10,939	84	2.32 (1.73, 3.12)	2.35 (1.71, 3.22)^*^
Comoros	3222	13	1.44 (0.86, 2.41)	1.45 (0.86, 2.44)
Madagascar	12,524	162	3.17 (2.42, 4.15)	3.21 (2.44, 4.22)^*^
Malawi	17,217	178	2.36 (1.81, 3.08)	2.13 (1.61, 2.80)^*^
Mozambique	11,612	92	2.18 (1.62, 2.94)	2.25 (1.65, 3.07)^*^
Rwanda	7881	122	4.07 (3.07, 5.41)	3.22 (2.41, 4.31)^*^
Tanzania	9891	161	4.94 (3.81, 6.42)	4.89 (3.76, 6.36)^*^
Uganda	15,040	230	3.91 (3.03, 5.04)	4.10 (3.17, 5.30)^*^
Zambia	9745	96	2.75 (2.06, 3.68)	3.20 (2.38, 4.31)^*^
Zimbabwe	6374	44	1.99 (1.39, 2.86)	2.13 (1.47, 3.08)^*^
**Maternal age**				
15–24	41,168	515	1	1
25–34	65,278	792	0.98 (0.88, 1.09)	1.27 (1.11, 1.45)^**^
≥ 35	30,785	265	0.76 (0.65, 0.87)	1.19 (1.01, 1.44)^*^
**Maternal education**				
No	33,056	392	1	1
Primary	72,912	898	1.01 (0.89, 1.13)	0.91 (0.80, 1.04)
Secondary or above	31,263	281	0.76 (0.65, 0.88)	0.68 (0.56, 0.82)*
**Respondent occupation status**				
No	44,237	380	1	1
Yes	92,995	1192	1.31 (1.17, 1.46)	1.09 (0.96, 1.23)
**Wealth status**				
Rich	48,396	492	1	1
Middle	26,494	326	1.17 (1.02, 1.35)	1.09 (0.93, 1.27)
Poor	62,341	735	1.08 (0.96, 1.21)	1.07 (1.02, 1.23)^*^
**Media exposure**				
yes	48,162	538	1	1
No	89,070	1032	1.29 (1.23, 1.36)	1.11 (1.01, 1.25^*^
**Marital status**				
Married	117,235	1378	1	1
Single	6450	32	2.43 (1.72, 3.44)	3.24 (2.27, 4.63)^**^
Divorced/widowed/separated	13,547	161	2.39 (1.64, 3.49)	2.99 (2.04, 4.39)^*^
**Place of delivery**				
Home	37,654	350	1	1
Health facility	99,577	1221	1.25 (1.12, 1.41)	1.05 (0.91, 1.21)
**Parity**				
1	21,804	369	1	1
2–4	70,872	838	1.42 (1.31, 1.54)	0.72 (0.62, 0.84)^**^
≥ 5	44,558	343	1.77 (1.63, 1.92)	0.43 (0.34, 0.53)^*^
**Number of ANC visit**				
No	6069	58	1	1
1–3	39,312	479	1.48 (1.28, 1.71)	0.91 (0.68, 1.22)
≥ 4	91,850	1035	1.50 (1.30, 1.73)	0.89 (0.67, 1.18)
**Mode of delivery**				
Vaginal	129,345	1413	1	1
Caesarean section	7887	158	1.35 (1.24, 1.48)	1.81 (1.52, 2.15)^*^
**Preceding birth interval (in months)**				
25–48	58,023	568	1	1
< 24	18,828	134	1.19 (1.10, 1.28)	1.15 (1.06, 1.24)^*^
≥ 49	60,380	869	1.06 (0.99, 1.15)	1.45 (1.28, 1.65)^**^

^*^p-value < 0.05, ** p-value < *0.01, COR: Crude Odds Ratio, AOR: Adjusted Odds Ratio, CI: Confidence Interval*

## Discussion

The overall aim of this study was to investigate the pooled prevalence and associated factors of stillbirth in East Africa using the recent DHS surveys conducted in 12 East African countries. The study demonstrated several socio-demographic and maternal obstetric and health service-related factors were significantly associated with stillbirth.

Our study reported that the prevalence of stillbirth in East Africa was 0.86% (95% CI: 0.82, 0.91). This was lower than studies reported in developing countries [36], a systematic review conducted worldwide [4], and the ENAP [37], it could be because East African countries have a lack of adequate access to obstetric care to manage maternal infections and complications like antepartum bleeding and pregnancy-induced hypertensive disease during labor and delivery. This study revealed that country, maternal age, media exposure, wealth status, marital status, mode of delivery, and preceding birth interval were significantly associated with stillbirth. Stillbirth was found to be higher older women. This was consistent with studies conducted in Zambia [38] and Australia [39], it might be due to advanced maternal age is strongly related to chronic medical and obstetrical conditions that may influence pregnancy outcomes and might increase the risk of experiencing stillbirth [40, 41]. Besides, as a woman gets older, both mothers and babies face an increased risk of pregnancy-related complications such as preeclampsia, gestational thromboplastin disease, twin gestations, and change in the reproductive system like increased risk of Down syndrome that could increase the risk of experiencing stillbirth [17, 42, 43]. Pregnant women in poor household wealth status were more likely to have to experience stillbirth than women in a rich household. It was supported by previous studies conducted in Uganda [44], and Nepal [45]. Wealthier mothers are more conscious about maternal health, the importance of a balanced diet, and the need for ANC visits, and maybe more likely to be aware of unhealthy behaviors [46]. Women in poor households are less likely to use maternal health care services such as family planning service, ANC visit, and health facility delivery though these services are free of charge they are unable to cover the indirect costs [47].

Media exposure decreases the odds of experiencing stillbirth. It was consistent with previous study findings [48–50], it might be due to mass media is the most powerful tool for increasing awareness and knowledge, and behaviors towards maternal health care service utilization [51, 52]. Exposure to mass media among reproductive-age women increased their use of, family planning services, ANC services, and health facility delivery therefore it can reduce the risk of stillbirth [53, 54]. Mothers who attained secondary education or above were less likely to experience stillbirth compared to mothers who didn’t have formal education. The possible justification could be due to maternal education could lead to the corresponding improvement in health-seeking behavior such as the timely decision to seek care appropriate care during pregnancy, give better care for their health and their fetus, awareness to the danger sign of pregnancy and maternal health service utilization.

In this study, maternal marital status was significantly associated with experiencing stillbirth. Birth from single/divorced/widowed women was at increased risk of stillbirth. It was supported by previous study findings [28, 55], the possible explanation might be due to unmarried women generally has significant stress and financial burden for using maternal health care services and unable to afford the indirect costs such as cost for transportation [56]. Besides, unmarried women lack social support and more likely to experience distress this could increase the risk of experiencing stillbirth [57]. Births through caesarean section had an increased risk of stillbirth than births delivered vaginally. It was consistent with studies reported in Nigeria [58] and Gambia [59]. This might be because in developing countries maternal health services were not available and reachable, particularly caesarean section is done at tertiary hospitals. Though caesarean section is applied to save the life of new-born in high-risk pregnancies. In East Africa, the majority of the population are rural residents and hospitals are not accessible due to transportation problems which resulting not saving the fetus's life because the caesarean section is not done at the right time. Therefore, high-risk deliveries like birth asphyxia, mal-presentation, fetal stress, and Antepartum Haemorrhage (APH) that needs caesarean delivery are referred from health centers and health posts and may not reach the right time to conduct caesarean section. This could increase the risk of stillbirth [60, 61]. Birth interval of < 24 months and > 48 months were associated with increased odds of stillbirth than births with the birth interval of 24–48 months. It was in line with study findings in India [18], and Swine [62], this could be explained by women who had short preceding birth interval are less able to provide nourishment for the fetus because her body had less time to recuperate from the previous pregnancy, and the uterus had less time to recover. Furthermore, lactation will deplete maternal nutrition and may end up with poor pregnancy outcomes [63]. Moreover, pregnant mothers who had birth interval of four years or above are more likely at risk of antepartum haemorrhage, and risk of medical complication as equivalent with primigravida and this could increases the risk of experiencing stillbirth.

These results should be interpreted in light of the following limitations. First, this study was based on DHS which was primarily collected to generate health and health-related indicators and therefore, important variables such as maternal underlying medical and obstetrical conditions like DM, HTN, cardiac problem HIV/AIDS, preeclampsia, gestational DM, and antepartum haemorrhage were not collected in DHS. In addition, the EDHS survey did not incorporate clinically confirmed data rather it relied on mothers' or caregivers' verbal autopsy and might have the possibility of social desirability bias [64]. Despite the abovementioned limitations, this study had several strengths. First, the study was based on weighted data to make it representative and it can be generalized to all births within the study period. Besides, this study was a pooled analysis that could increase the study power to permit a full examination of effect modification within the data and can reduce the measurement errors and bias arising when studies are combined that used heterogeneous designs and data collection methods. The findings of this study have valuable policy implications for health program design and interventions. Stillbirth high-risk areas can be easily identified to make effective local interventions.

## Conclusion

Our study found that the stillbirth rate remains a major public health problem in East Africa and it was far below to achieve the ENAP target by 2030. Maternal age, maternal education status, mode of delivery, preceding birth interval, marital status, media exposure, and wealth status were significantly associated with experiencing stillbirth. These findings highlight the weak health care system of East African countries. Therefore, public health programs should be designed for enhancing adequate birth spacing, and media access to the community to reduce the incidence of stillbirth.

## Data Availability

All relevant data related to the study were included in the manuscript. The datasets used for the analysis of the study can also be obtained after the reasonable request of the DHS Program using the link https://dhsprogram.com/data/dataset_admin/index.cfm.

## Abbreviations

ANC: Antenatal Care
AOR: Adjusted Odds Ratio
ARR: Annual Rate of Reduction
BMI: Body Mass Index
CI: Confidence Interval
COR: Crude Odds Ratio
CSA: Central Statistical Agency
DHS: Demographic Health Survey
EA: Enumeration Area
EDHS: Ethiopian Demographic Health Survey
GIS: Geographic Information System
ICC: Intra-cluster Correlation Coefficient
IUFD: Intra Uterine Fetal Death
IUGR: Intra Uterine Growth Restriction
LLR: Log-likelihood Ratio
LR: Likelihood Ratio
PHC: Population and Housing census
SBR: Stillbirth Rate
SNNPRs: Southern Nations and Nationality People Regional state
WHO: World Health Organization
